# Microstructure and Abrasive Wear Resistance of Metal Matrix Composite Coatings Deposited on Steel Grade AISI 4715 by Powder Plasma Transferred Arc Welding Part 2. Mechanical and Structural Properties of a Nickel-Based Alloy Surface Layer Reinforced with Particles of Tungsten Carbide and Synthetic Metal–Diamond Composite

**DOI:** 10.3390/ma14112805

**Published:** 2021-05-25

**Authors:** Artur Czupryński

**Affiliations:** Welding Department, Faculty of Mechanical Engineering, Silesian University of Technology, Konarskiego 18A, 44-100 Gliwice, Poland; artur.czuprynski@polsl.pl

**Keywords:** PPTAW, cladding, deposition, abrasion, impact load, tungsten carbide, synthetic metal–diamond composite

## Abstract

The article is the continuation of a cycle of works published in a Special Issue of MDPI entitled “Innovative Technologies and Materials for the Production of Mechanical, Thermal and Corrosion Wear-Resistant Surface Layers and Coatings” related to tests concerning the microstructure and mechanical properties of innovative surface layers made using the Powder Plasma Transferred Arc Welding (PPTAW) method and intended for work surfaces of drilling tools and machinery applied in the extraction industry. A layer subjected to tests was a metal matrix composite, made using powder based on a nickel alloy containing spherical fused tungsten carbide (SFTC) particles, which are fused tungsten carbide (FTC) particles and spherical particles of tungsten-coated synthetic metal–diamond composite (PD-W). The layer was deposited on the substrate of low-alloy structural steel grade AISI 4715. The results showed that the chemical composition of the metallic powder as well as the content of the hard phase constituting the matrix enabled the making of a powder filler material characterised by very good weldability and appropriate melting. It was also found that the structure of the Ni-WC-PD-W layer was complex and that proper claddings (characterised by the uniform distribution of tungsten carbide (WC)) were formed in relation to specific cladding process parameters. In addition, the structure of the composite layer revealed the partial thermal and structural decomposition of tungsten carbide, while the particles of the synthetic metal–diamond composite remained coherent. The deposited surface layer was characterised by favourable resistance to moderate dynamic impact loads with a potential energy of 200 J, yet at the same time, by over 12 times lower metal–mineral abrasive wear resistance than the previously tested surface layer made of cobalt-based composite powder, the matrix of which contained the hard phase composed of TiC particles and synthetic metal–diamond composite. The lower abrasive wear resistance could result from a different mechanism responsible for the hardening of the spherical particles of the hard phase susceptible to separation from the metal matrix, as well as from a different mechanism of tribological wear.

## 1. Introduction

Most plasma cladding applications involve the use of a powder filler material enabling the obtainment of deposited layers characterised by various chemical compositions, structure and properties [1,2]. In deposited layers that have the structure of composite materials (used to improve the abrasive wear resistance of drilling tools or machinery), the matrix is usually composed of cobalt, nickel or iron-based alloys containing particles of high-melting phases, e.g., carbides of transition metals found in groups IVB–VIB of the periodic table [3,4]. High hardness, high melting points and high thermodynamic stability constitute the primary and very desirable features of the aforesaid carbides [5]. The combination of hard carbide phases with the relatively plastic metal matrix significantly improves the functional properties of surface layers exposed to various types of abrasion. Composite layers are characterised by particularly high abrasive wear resistance as well as resistance to moderate impact loads, unobtainable in cases of typical metallic layers. Under operational conditions of drilling tools (discussed in detail in the first part of the cycle of publications), it appears particularly reasonable to use composite layers reinforced with tungsten carbide particles [6,7].

Deposited composite layers are characterised by the repeatable structure providing high operational properties, particularly when particles of the hardening phase are uniformly distributed in the metal matrix. There are many factors affecting the formation of deposited composite layers and the dispersion of particles in the hard reinforcing phase. The most important of the aforementioned factors include the nature and the intensity of the effect of the liquid metal matrix containing the particles of the hardening phase (indicated by the wetting degree). According to Bober et al. (2016) [8], good wettability is observed when both the liquid and the solid phase are characterised by the same type of atomic bonds and when appropriate temperature conditions and surface activation conditions are satisfied. The carbides of the transition metals of the periodic table such as Ti, Zr, Nb, Cr, Hf, Nb, Ta, Mo and W are characterised by the complexity of chemical bonds. Typically, the above-named carbides contain mixed bonds, i.e., metallic bonds with the covalent-type effect or, in certain cases, with the ionic effect [9]. During the cladding process, when the particles of the hard phase characterised by a high melting point are wetted appropriately by the liquid metallic matrix, proper welds are formed and high yield (degree of carbide powder use) is obtained. In cases of insufficient wettability, the particles of the reinforcement (hardening) are expelled from the melt pool and the yield of hardening phase particles is low. The above-named situation may result in the formation of many welding imperfections including cracks, gas pores or the internal porosity of the deposited layer. In addition to providing composite components with good wettability, the difference in density between the hard phase and the matrix favourably affects the formation of composite claddings. Regarding the metal of the matrix and that of the hardening phase, significantly varying mass densities may result in the non-uniform distribution or the agglomeration of the hard phase particles in the volume of the cladding metal. Carbides characterised by high specific density tend to settle to the bottom of the melt pool, whereas the particles of the hard phase with lower density concentrate in the upper zone, slightly below the cladding weld. Many research works are concerned with the thorough investigation of the structure and the abrasive wear resistance of deposited composite layers that have the nickel alloy-based matrix containing the addition of tungsten carbide [10,11], chromium carbide [12,13] and titanium carbide [4,14,15]. However, significantly fewer publications focus on composite claddings reinforced with particles of the remaining carbides of transition metals, e.g., ZrC, HfC, NbC, TaC and MoC, or synthetic metal–diamond sinters. As the aforementioned reinforcement particles are also characterised by advantageous properties, their addition could significantly improve the abrasive wear resistance of deposited layers [16]. In light of research results presented in the first part of the cycle of publications, the use of the hard phase particles in the form of synthetic metal–diamond composite seems very promising. Powder filler materials that could be used in the PPTAW metal deposition of composite layers on selected structural materials are presented in Table 1.

Metal matrix composite (MMC) layers reinforced with, e.g., TiC, WC, B_4_C, Cr_3_C_2_, NbC, etc. particles, combine the properties of the plastic, abrasive wear and corrosion resistant matrix with the properties of hard carbide ceramics [27]. Surface layers of the aforesaid type can be used where it is necessary to ensure high abrasive wear resistance combined with resistance to dynamic impact loads. Such requirements cannot be satisfied by commonly used metallic layers applied using welding methods or by hard and brittle ceramic layers. Therefore, it seems favourable to use MMC layers deposited on work surfaces of drilling tools. The addition of hard carbide particles characterised by the various degree of dispersion additionally reinforces the matrix. According to Deuis et al. (1997) metal matrix composites can transfer higher compressive and tensile stresses than monolithic alloys. This is because applied loads are transferred from the plastic matrix to the particles of the hardening phase, which is possible if there are appropriate bonds between individual components of a given composite [26]. During the deposition of composite layers, the nature of the interphase boundary between the metal matrix and the hard reinforcing phase depends on the time and the temperature of cladding formation as well as on the chemical composition of the matrix. The growing popularity of these materials also results from their advantageous structural ratio, i.e., the proportion of strength to mass. The acceptable quality and the highly satisfactory structural and mechanical properties of the composite layer with the composite powder based on a cobalt alloy containing titanium carbide (TiC) and synthetic metal–diamond composite [14] inspired the author’s research on the material characteristics of the layer made of composite powder based on nickel with the addition of hard reinforcing phase composed of fused tungsten carbide (WC) and synthetic metal–diamond sinter. There is no scientific information and material data related to the abrasion tests concerning metal matrix surface layers reinforced with synthetic metal–diamond composite and obtained using the Powder Plasma Transferred Arc Welding (PPTAW) metal deposition method. The results of previous individual research led to the general conclusion that it was possible to obtain a nickel-based alloy surface layer reinforced with particles of tungsten carbide and synthetic metal–diamond composite that can characterize a good resistance to abrasive wear.

## 2. Experimental Section

### 2.1. Objective of the Study

The study aimed to assess the metallographic structure as well as to identify the metal–mineral abrasive wear resistance and the resistance to moderate impact loads of a composite layer deposited using the PPTAW metal deposition method and “newly developed” nickel alloy-based powder with the addition of the hard reinforcing phase containing two types of tungsten carbide (WC-W_2_C) and synthetic metal–diamond composite (PD-W). It was assumed that the spherical shape of the hard phase particles would result in their firm deposition in the metallic matrix, thus significantly improving the abrasive wear resistance parameters of the deposited layer protecting work surfaces of drilling tools used in the extractive industry. The results of the relative abrasive wear resistance of the nickel alloy-based composite layer were compared with those concerning the cobalt-based surface layer [22]. Criteria specifying the requirements related to the making and the acceptance of the deposited layer are presented in detail in the first part of the cycle of publications.

### 2.2. Materials and Methods

**Figure 1:**
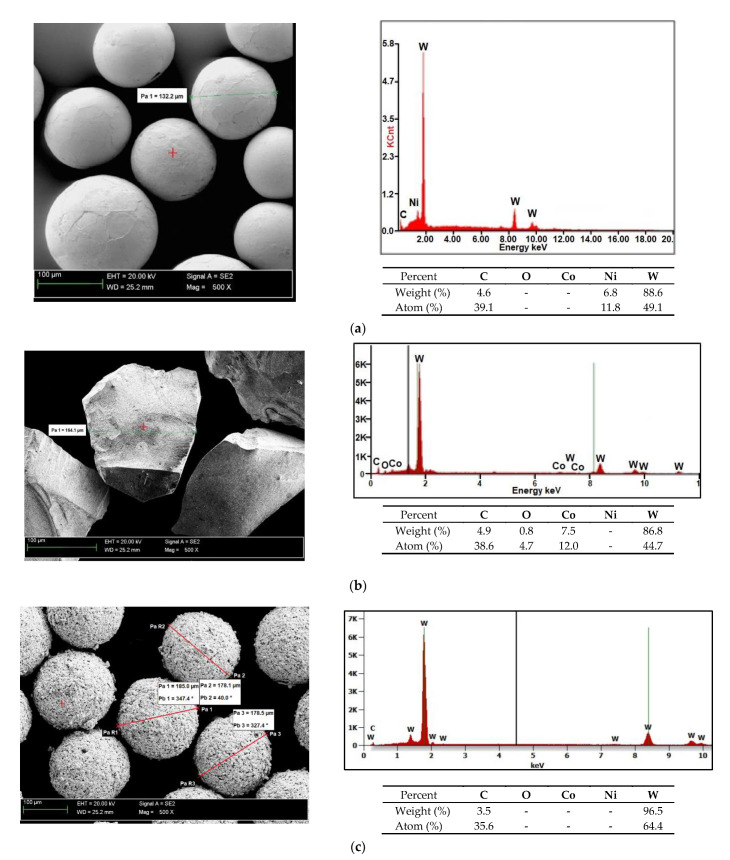
Components of the hard ceramic phase in the Ni3+WC-W_2_C+PD-W powder: (**a**) spherical fused tungsten carbide (SFTC), (**b**) fused tungsten carbide (FTC) and (**c**) tungsten-coated synthetic metal–diamond composite (PD-W).

The surface layer was deposited on the substrate of low-alloy structural steel AISI 4715 (Table 2) having the form of a flat bar (75 mm × 25 mm × 10 mm). The surface of the substrate was cleaned directly before the cladding process. The filler material used in the process was a powder mixture, the matrix of which was a nickel alloy having a chemical composition corresponding to that of the group of Ni3 alloys (in accordance with EN 147000:2014) [28] (Table 2). The powder mixture also contained a hard phase composed of spherical fused tungsten carbide (SFTC) particles (Figure 1a), fused tungsten carbide (FTC) particles (Figure 1b) and spherical particles of tungsten-coated synthetic metal–diamond composite (PD-W) (Harmony Industry Diamond, Zhengzhou, China) (Figure 1c). The particles of the WC-W_2_C tungsten carbide and synthetic metal–diamond composite (PD-W) were mixed with the matrix powder in the 60/35 wt% ratio using a mixer-shaker and ceramic balls.

**Figure 2:**
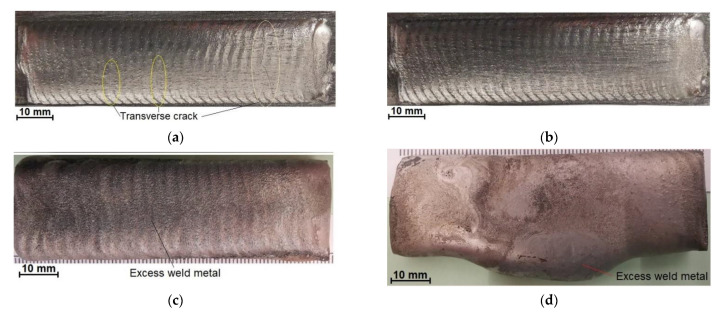
Optimization process parameters of the PPTAW metal deposition of the surface layer (Ni3+WC-W_2_C+PD-W composite powder) on steel AISI 4715: (**a**) too low heat input (275 J/mm); (**b**) the right heat input (400 J/mm); (**c**) too much heat input (520 J/mm); (**d**) too much heat input (580 J/mm) and amount of supplies powder (22 g/min).

The composite layer was deposited using an industrial welding station provided with a modern robotic hardfacing plasma coating machine for cladding with PPTAW Durweld 300T PTA (Durum Verschleiss-Schutz GmbH, Willich, Germany) having a maximal current of 300 A and parameters determined during initial cladding tests (Figure 2, Table 3). The deposited layer was obtained by weave-bead technique with an overlap of 33%. 

The cladding parameters identified as optimum were those ensuring the uniform distribution of the powder over the entire liquid metal area in the weld metal pool, uniform and shallow penetration having depth of g = 1.2 mm, the height of the layer in one run h ≥ 2 and the dilution of the base material in the cladding D below 4.5%.

### 2.3. Testing Methodology

The testing methodology and equipment are discussed in detail in the first part of the cycle of publications. The tests involving the analysis of the morphology and the size of the composite powder (MMC) particles as well as the assessment of the quality of the layer were based on non-destructive tests including visual tests (VT), penetrant tests (PT) and tests concerning the roughness (Ra) of the composite layer. The analysis of the structure and of the surface properties of the composite layer was based on macro and microscopic metallographic test results, chemical composition analysis results, X-ray diffraction results, weld deposit hardness and roughness measurement results, as well as results of tests concerning metal–mineral abrasive wear resistance and resistance to moderate impact loads.

## 3. Results and Discussion

### 3.1. Composite Powder Morphology

**Figure 3:**
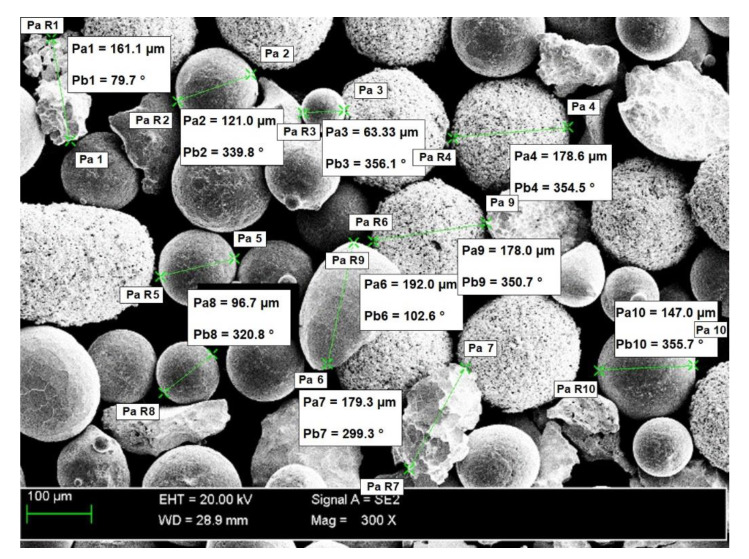
Scanning electron microscopic (SEM) image of the morphology of the Ni3+WC-W_2_C+PD-W powder particles along with measurement values.

The morphology and the size of the Ni3+WC-W_2_C+PD-W powder particles are presented (in the form of SEM images) in Figure 3. The image of the powder mixture components was made in the contrast of back-scattered electrons (BSE); the bright particles represent the tungsten carbide particles, whereas the darker particles are the nickel alloy particles. The tests revealed that the size of the powder particles was restricted within the range of 30 μm to 170 μm (median Q_50_ = 152 μm) and that the powder was a homogenous mixture containing primarily spherical components and a small fraction of irregularly-shaped particles. The components of the powder were mixed in a laboratory powder mixer-shaker, using ceramic balls. The powder mixer-shaker of a container with two perpendicular rotational axes was used. Main rotational axis was driven with constant rotational velocity of 46 rpm. The secondary axis was performing rocking motions in the range 0–180°. The powder ingredients were mixed for 1 minute. The particle bulk were moving across the vessel through a three-dimensional unsteady periodical behaviour.

**Figure 4:**
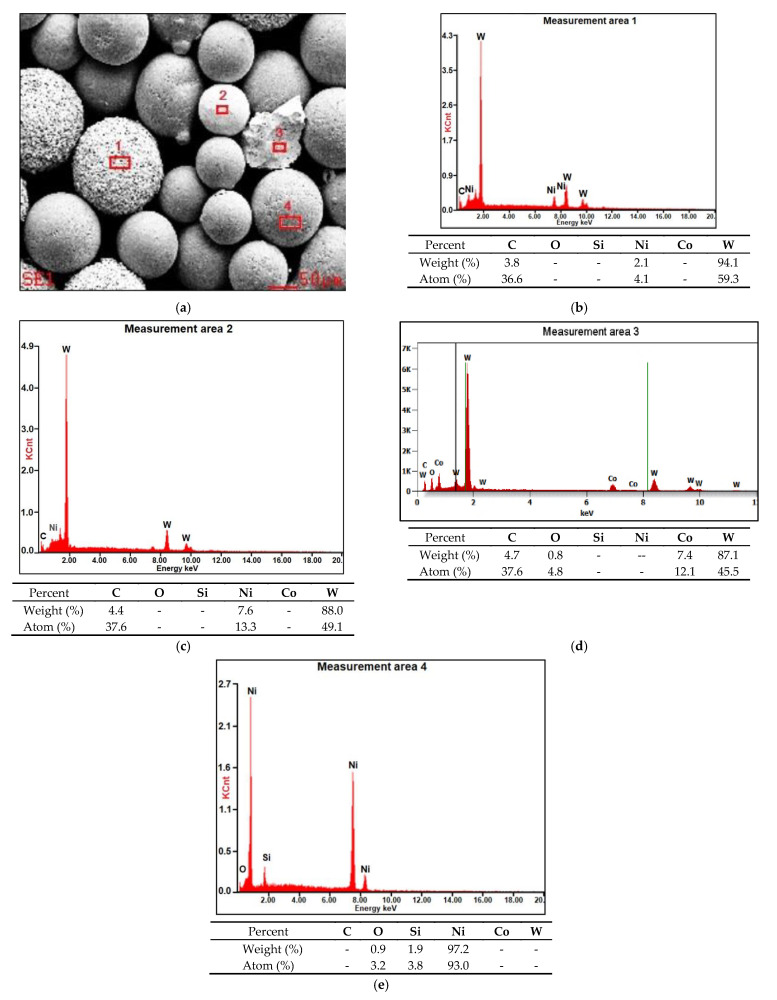
Morphology, the size of the powder mixture particles and the microanalysis results related to the chemical composition of the Ni3+WC-W_2_C+PD-W composite powder: (**a**) SEM image of the morphology of the powder particles with the area subjected to analysis and the diagrams of scattered X-radiation energy with energy lines present in the area of components (chemical elements) subjected to analysis; (**b**) tungsten-coated synthetic metal–diamond composite (PD-W); (**c**) spherical fused tungsten carbide (SFTC); (**d**) fused tungsten carbide (FTC); (**e**) nickel matrix.

The applied manner of mixing favourably limited the agglomeration of components and enabled the obtainment of the powder filler material characterised by good flowability. Microanalysis results concerning the chemical composition of the powder are presented in Figure 4 (in the form of photographs and diagrams of scattered X-radiation). The tests were performed on the surface of the powder particles using point or micro-area-based analysis.

### 3.2. Non-Destructive Tests—Visual Test Results

**Figure 5:**
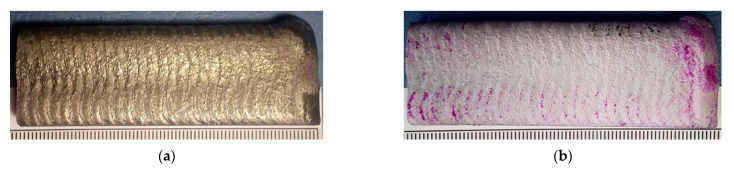
Surface layer made using the Ni3+WC-W_2_C+PD-W composite powder: (**a**) layer after the visual tests (VT); (**b**) layer after the penetrant tests (PT).

The visual and the penetrant tests of the surface of the deposited surface layer only revealed the presence of apparent welding imperfections or single gas pores (Figure 5b). The above-named tests did not reveal shape and dimension-related imperfections. The average surface roughness (Ra) amounted to 12 μm. Roughness tests were performed in five test lines on the untreated surface. Comparing the obtained test results with the results presented in [22], it should be stated that the average surface roughness of the composite layer on the matrix of the nickel alloy was slightly lower (about 2 μm) than the roughness of the layer on the matrix of the cobalt alloy. The overlapping beads were characterised by appropriate symmetry, which translated into the uniform distribution of the composite powder on the specimen surface (Figure 5a).

In view of the fact that the surface layer is intended for work surfaces of drilling tools, the presence of single small gas pores on the surface of the deposited layer can be regarded as acceptable.

### 3.3. Metallographic Test Results and Results of the XRD Analysis

**Figure 6:**
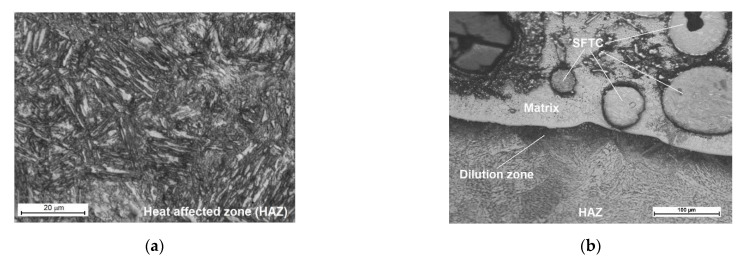

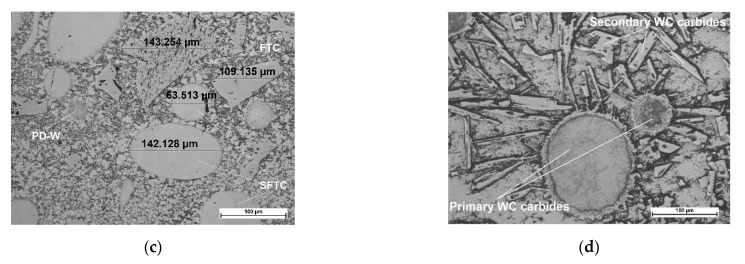
Microstructure of the surface layer (PPTAW metal deposition method, Ni3+WC-W_2_C+PD-W composite powder) deposited on structural low-alloy steel AISI 4715: (**a**) heat-affected zone (HAZ); (**b**) dilution zone; (**c**) size and distribution of the hard phase in the middle of the cladding; (**d**) distribution of the reinforcing phase near the padding weld.

**Figure 7:**
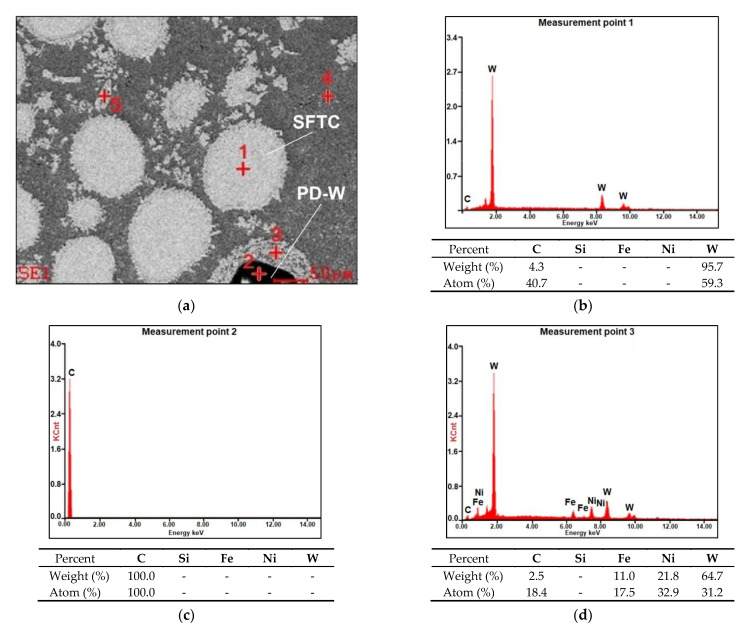

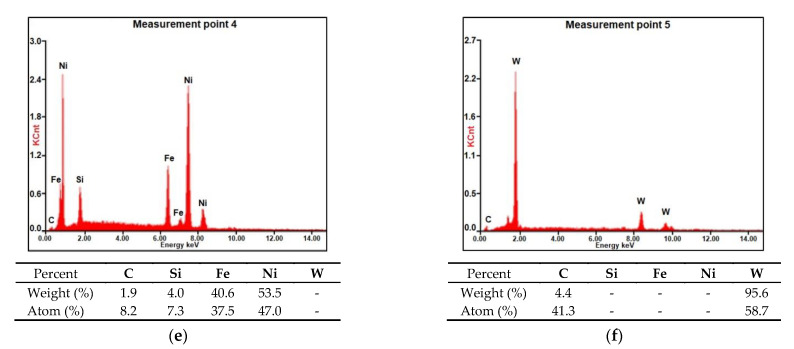
Microstructure and the results of the qualitative point analysis in the carbide–matrix area of the surface layer (PPTAW metal deposition method, Ni3+WC-W_2_C+PD-W composite powder) deposited on structural low-alloy steel AISI 4715: (**a**) area subjected to analysis; (**b**–**f**)diagram of the energy of scattered X-radiation with energy lines present in the area of components (chemical elements (ceramic particle of WC, synthetic metal–diamond composite and metal matrix)) subjected to analysis.

The results of microscopic metallographic observations made it possible to identify the structure of the matrix as well as the type, distribution and the dimensions of the surface layer reinforcement. The results of the microstructural observations of the surface layer cross-section are presented in Figure 6. The microstructure and the results of the qualitative point analysis, identifying the individual chemical elements present in the surface layer (made using the PPTAW method), are presented in Figure 7.

The microstructure of the layer was composed of spherical particles of primary tungsten carbides in the nickel alloy matrix. The carbides were present in the entire area of the deposited layer. The carbides were mostly uniformly distributed in the layer, without forming agglomerates typical of ceramic composite castings with the metal matrix [29]. The uniform distribution of carbides in the surface layer resulted from the proper blending of the powder mixture and the fact that the density of tungsten carbide was only twice that of the matrix. During the cladding process, the use of mixtures composed of ingredients characterised by significantly varying density may lead to the formation of agglomerates of the hard reinforcing phase, the reduction of their wettability by the matrix alloy and to the formation of welding imperfections [3,11]. The microstructure of the substrate in the heat-affected zone was composed of martensite or tempered martensite (Figure 6a). In turn, the microstructure of the matrix contained dendritic grains, on the boundary of which it was possible to observe eutectics (Figure 6c). The quantitative analysis performed in measurement points marked in Figure 7a in the carbide–matrix area revealed that the chemical composition in measurement points 1 and 5 corresponded to that of tungsten carbide. Measurement point 2 contained synthetic metal–diamond composite, the tungsten coating of which (measurement point 3), after partial melting, was responsible for the transfer of tungsten to the solution of the composite matrix (alloy Ni-C-Fe-Si). The presence of iron in the composite matrix resulted from the dilution of the base metal in the layer, amounting to approximately 4.5%. The cladding process was accompanied by the complete melting of the nickel alloy powder (T_melt._ of approximately 1300 °C) and the partial melting of the primary tungsten carbides (T_melt._ = 2870 °C), potentially leading to the formation of complex secondary carbides on the carbide–matrix boundary [11]. The secondary carbides were responsible for the diffusive bond of the primary carbides with the matrix. According to Bober et al. (2011) [3] and Poloczek et al. (2019) [10], the above-named mechanism of the bonding of carbides with the matrix should ensure their stable deposition. The partial melting of the primary carbides led to the partial saturation of the matrix with tungsten and carbon (Figure 7b).

**Figure 8:**
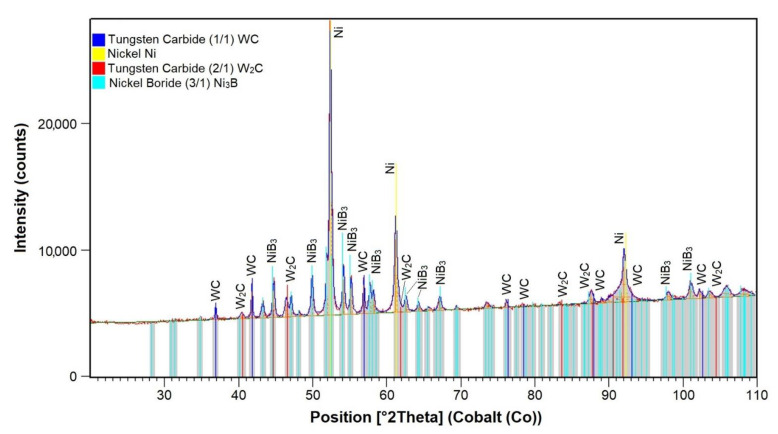
XRD pattern of the surface layer (PPTAW metal deposition method, Ni3+WC-W_2_C+PD-W composite powder) deposited on structural low-alloy steel AISI 4715 with the standard lines of identified crystalline phases.

The structural X-ray diffraction analysis aimed to identify phases present in the composite layer. An exemplary XRD pattern is presented in Figure 8, whereas the results of the X-ray qualitative phase analysis are presented in Table 4. The analysis revealed the presence of the γ-Ni solid solution and of the γ-Ni/Ni_3_B eutectic phase, which was consistent with information provided in related reference publications [30,31]. In addition, the structure also contained hexagonal carbide having the W_2_C structure as well as hexagonal carbide having the WC structure.

### 3.4. Density and Porosity of the Deposited Layer

**Figure 9:**
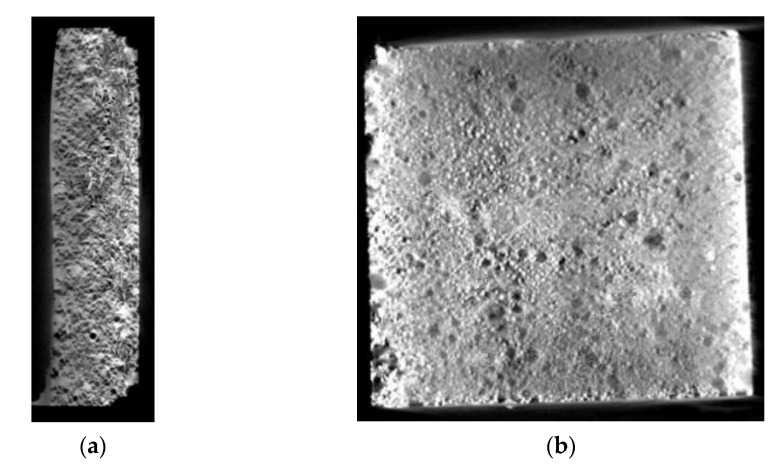
Images obtained as a result of the µCT analysis of the surface layer (laser metal deposition method, Ni3+WC-W_2_C+PD-W composite powder) deposited on structural low-alloy steel AISI 4715: (**a**) specimen porosity in cross-section surface; (**b**) specimen porosity in longitudinal section surface.

The density, porosity and absorbability of the composite was determined with the Archimedes method (according to ISO ASTM-D-792) on the basis of measurements concerning the mass of a specimen sampled from the surface layer. The apparent density was determined using a pycnometer. The pycnometer was used in ISO 1183–1:2004 standard. The results of related measurements and calculations are presented in Table 5. The surface layer porosity was analysed using a µCT microtomography. The images obtained as a result of the µCT analysis of the composite layer are presented in Figure 9.

The composite layer was characterised by a relatively high total porosity of more than 11% and a density of 9.34 g/cm^3^. The total porosity of the tested composite layer was almost twice as high as the porosity of the layer made of cobalt alloy reinforced with TiC and synthetic metal–diamond composite particles [22]. According to Bober et al. [19] (2018), this may be due to the poor wettability of the hard reinforcement phase by the nickel alloy matrix, especially in the case of bigger grains. In respect of the fact that the composite layer is intended for work surfaces of drilling tools, such high porosity can appear problematic and requires the performance of in situ tests involving the use of an actual tricone bite.

### 3.5. Hardness Measurements Test Results

**Figure 10:**
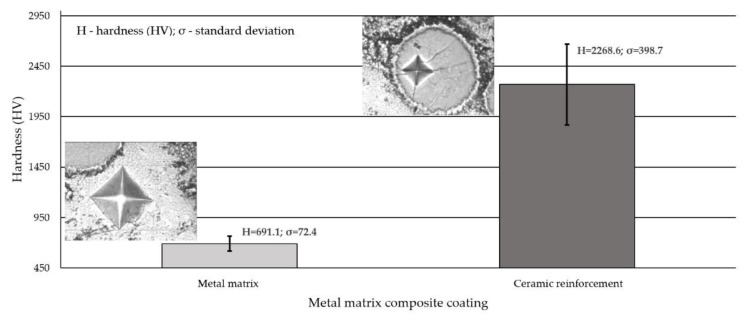
Hardness (HV0.5) measurement results concerning the cross-section of the surface layer (PPTAW metal deposition method, Ni3+WC-W_2_C+PD-W composite powder) deposited on structural low-alloy steel AISI 4715.

The results of hardness measurements concerning the external surface and the cross-section of the deposited layer are presented in Table 6 and Figure 10, respectively.

Hardness measured on the external surface of the deposited layer was slightly above 700 HV10 (approximately 60 HRC) and was approximately 208 HV10 higher than the hardness of the reference material used in abrasive wear tests, i.e., abrasion-resistant steel AR400. The average microhardness of the matrix of the composite cladding (obtained using the powder plasma transferred arc cladding method) measured on the cross-section of the layer at 10 measurement points amounted to 691 HV0.5 and was nearly the same as the hardness measured on the surface. The profile of the microhardness of the cross-section was sharp, which resulted from the composite structure of the cladding. The average hardness of ceramic reinforcement amounted to approximately 2270 HV0.5. The high value of the standard deviation resulted from the large discrepancy of the results between the hardness particles of the fused tungsten carbide (about 1400 HV0.5) and spherical fused tungsten carbide (about 2600 HV0.5). As the particles of synthetic metal–diamond composite has ultrahigh hardness compared to the hardest carbides currently used in the industry, the hardness of this matrix reinforcement was difficult to quantify. It was found that the length of the Vickers indentation on the compact surface of the polished synthetic metal–diamond composite was too small to be measured, even if we increased the load force to 9.8 N with a dwelling time of 15 s. Yahiaoui et al. (2016) [32] describe similar research difficulties when assessing hardness of graded polycrystalline diamond compact cutters. It was revealed that the hardness of the matrix increased along with the distance between the measurement point and the fusion line (growing towards the surface of the layer). Because of the dilution of the weld metal in the layer and due to the different cladding crystallisation manner, the microstructure of the layer in the area adjacent to the HAZ differed from the microstructure of the subsurface layer.

### 3.6. Abrasive Wear Test Results

**Figure 11:**
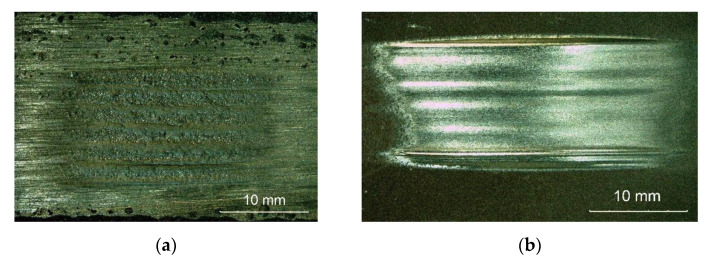
Surface of the composite layer and the surface of abrasion-resistant steel AR400 after the metal–mineral abrasive wear resistance test ASTM G64: (**a**) magnified area of layer abrasion; (**b**) magnified area of steel abrasion after the abrasive wear resistance test.

**Figure 12:**
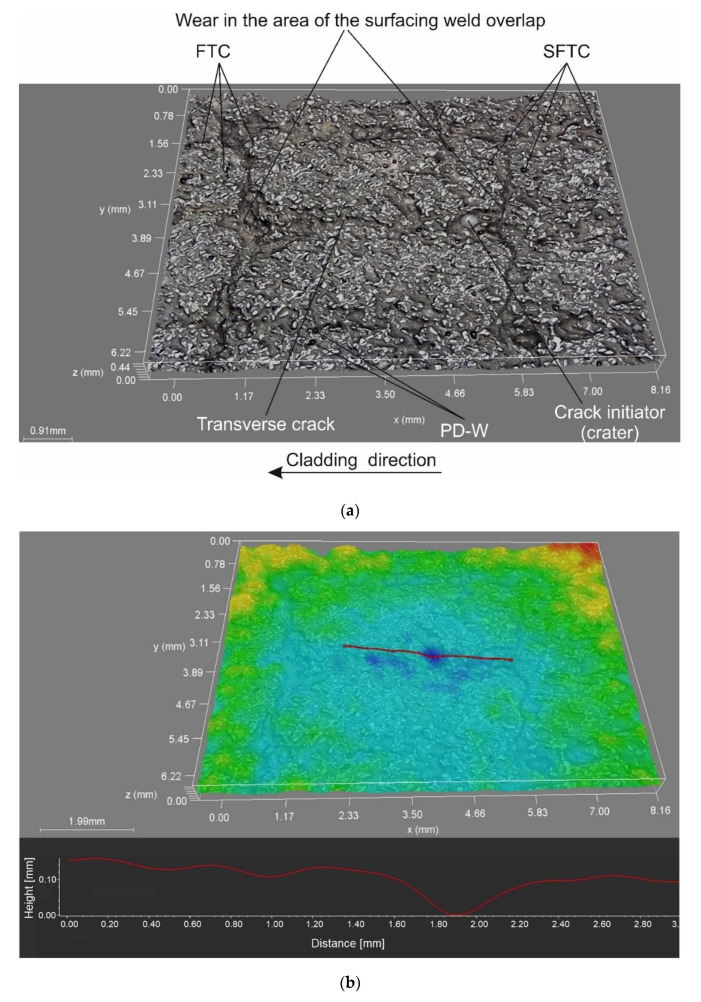

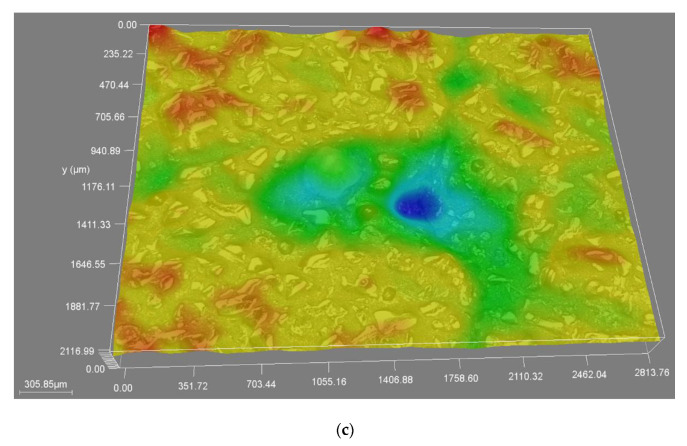
Surface of the composite layer after the metal–mineral abrasive wear resistance test observed using the confocal microscope: (**a**,**c**) main view of specimen wear; (**b**) measurement of defects (single craters).

The tests concerning the metal–mineral abrasive wear resistance of the surface layer were performed in accordance with ASTM G65, Procedure A, and referred to the abrasive wear resistance of a plate made of popular abrasion-resistant steel AR400 (made by a Swedish manufacturer) (SSAB AB, Stockholm, Sweden). The tests enabled the determination of the relative abrasive wear resistance of the surface layer (Table 7). The test results were compared with those obtained in relation to the previously tested Co3+TiC+PD-W composite layer. The nature of the abrasive wear of the surface layer was assessed on the basis of visual tests as well as observations involving the use of a stereoscopic microscope (Figure 11) and a confocal microscope (Figure 12).

**Figure 13:**
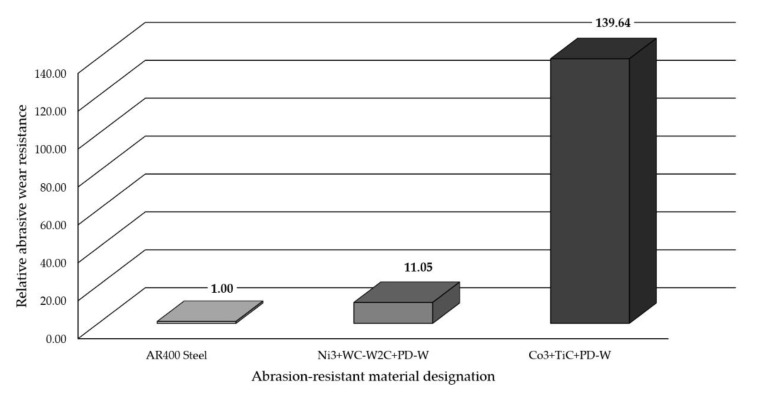
Relative metal–mineral abrasive wear resistance (ASTM 65-00, Procedure A) of the abrasive wear resistant deposited layers containing innovative cobalt and nickel alloys compared with the abrasive wear resistance of abrasion-resistant steel AR400.

The abrasive wear process, performed using the medium pressure of the counterspecimen affecting the surface of the deposited layer, led to the partial crushing of abrasive particles. The relative abrasive wear resistance of the deposited layer was higher than that of abrasion-resistant steel AR400 (slightly over 11 times). The average loss of surface mass after the test amounted to 0.1894 g. The assessment of the surface after the test revealed the abrasive mechanism of wear. The hard phase, having the form of spheroidal particles of tungsten carbide (WC) and synthetic metal–diamond composite (PD-W), did not reveal sufficient embedment in the nickel alloy-based matrix and was relatively easily peeled by the abrasive medium. The dominant wear mechanism affecting the surface layer was micro-cutting, manifested by the presence of continuous micro-scratches along the traces of wear and, to a significantly lesser extent, by the micro-ridging of the surface. The most intensive wear was observed in the overlap area (Figure 12a). A similar mechanism of increased wear of the layer deposited with nickel alloy in the area of overlapping seams was confirmed by Katsich et al. (2006) [6]. The authors state that the wear behaviour of WC-W_2_C reinforced Ni-based MMCs strongly depends on the formation of hard phase structures during manufacturing process. The surfacing parameters and the cladding bead trajectory are of particular importance. The abrasive grains uniformly affected the surface of the layer, where it was possible to notice small single craters after removing particles of the hard reinforcing phase. The micro-scratches were parallel in relation to the longer side of the specimen (which confirmed the thesis about the insufficient embedment of the particles of the carbide ceramics). Detailed examination involving the use of confocal microscopy revealed the presence of single crack (Figure 12a) and single crater (having a depth of up to 100 μm) inside the layer (Figure 12b,c). The abrasive wear process was significantly intensified by the effect of loose particles of the hard carbide phase torn out of the matrix and moving between the surfaces of the specimen and of the “rubber wheel”. The aforesaid situation could significantly increase the abrasive wear of the surface layer. The freely rolling spheroidal tungsten carbides (WC) and synthetic metal–diamond composite particles were primarily responsible for the plastic deformation of matrix fragments, manifested by the presence of characteristic micro-ridges. The aforementioned observation was confirmed by results obtained by Cheng et al. (2013) [33] who, in their work, mentioned such a possibility. The thesis that the partial surface melting of the primary tungsten carbides could translate into a sufficiently high increase in the diffusive force of the bond between the carbides and the matrix was not confirmed. In relation to data contained in previous publications [34] and individual research [12] concerning the abrasive wear resistance of surface layers made of nickel alloys reinforced only with the hard WC layer (SFTC), it was possible to notice the highly favourable abrasion-resistant effect of synthetic metal–diamond composite particles (PD-W), constituting approximately 20 wt% of the entire matrix reinforcement. The comparison of the abrasive wear resistance of the layer made using the Ni3+WC-W_2_C+PD-W composite powder with that of the layer made using the Co3+TiC+PD-W composite powder revealed that the abrasive wear resistance of the cobalt-based layer was more than 14 times higher (Figure 13).

The obtained test results concerning the abrasive wear resistance of the composite layer should be confirmed under actual conditions affecting the operation of drilling tools, including the ground structure, texture, the presence of stresses as well as hydrogeological conditions and humidity.

### 3.7. Impact Resistance Test Results

**Figure 14:**
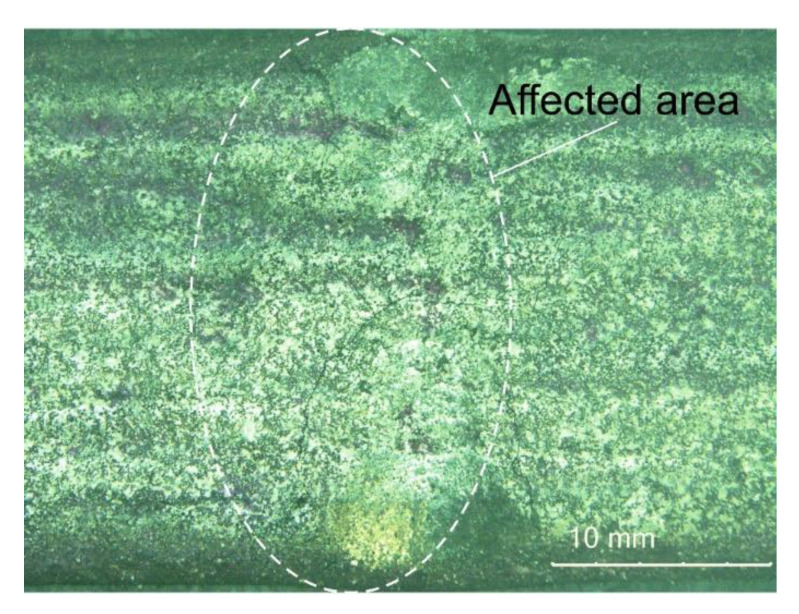
Condition of the surface of the composite layer (PPTAW metal deposition method, Ni3+WC-W_2_C+PD-W composite powder) deposited on structural low-alloy steel AISI 4715-after impact resistance tests.

The condition of the deposited layer surface during the individual stages of tests concerning resistance to moderate impact loads is presented in Figure 14.

The deposited layer made using the Ni3+WC-W_2_C+PD-W composite powder was characterised by highly favourable resistance to moderate dynamic impact loads. After a cycle of 20 (ram) strokes affecting the surface of the layer with a potential energy of 200 J, no damage in the form of visible cracks or chips of the weld deposit was observed. The only visible deformation was the plastic distortion of the deposited layer, particularly near the edges of the specimen.

## 4. Conclusions

The research-related tests aimed to assess the metallographic structure as well as to identify the metal–mineral abrasive wear resistance and the impact resistance of the innovative composite surface layer obtained using the Powder Plasma Transferred Arc Welding (PPTAW) metal deposition method and the Ni3+WC-W_2_C+PD-W powder. The analysis of the above-presented test results justified the formulation of the following conclusions:

1. The obtained layer was characterised by the classical composite structure and the uniform distribution of reinforcement composed of primary tungsten carbides (WC-W_2_C) and the particles of synthetic metal–diamond sinter in the matrix composed of the γ-Ni solid solution and the γ-Ni/Ni_3_B eutectic phase.
2. The applied powder plasma transferred arc (PPTAW) metal deposition method favours the maintaining of the structural and thermal stability of the particles of the ceramic reinforcement of the matrix having the form of tungsten-coated synthetic metal–diamond composite (PD-W). The partial surface melting of the primary spherical tungsten carbides (WC) did not significantly increase the force of the diffusive bond between the hard phase and the matrix. During the abrasion test, the spherical particles of the carbide reinforcement (WC) underwent peeling, likely because of the insufficient wetting of the particle surface with the metal of the matrix.
3. The absolute porosity of the composite layer slightly exceeded 11%, whereas its specific density amounted to 9.34 g/cm^3^. The average hardness of the composite matrix amounted to 691 HV0.5, whereas tungsten carbides were characterised by a hardness of approximately 2268 HV0.5.
4. The relative metal–mineral abrasive wear resistance of the deposited composite layer obtained using the Ni3+WC-W_2_C+PD-W powder was more than 11 times higher than that of abrasion-resistant steel AR400 and more than 14 times lower than the abrasive wear resistance of the layer obtained using the Co3+TiC+PD-W powder [22].
5. The very high resistance of the composite layer to moderate dynamic impact loads appears very promising as regards to its application as the preventive protection of work surfaces of drilling tools used in the extractive industry.

The third part of the article will contain comparative test results concerning the brittle fracture resistance of iron, nickel and cobalt-based composite layers.

## Figures and Tables

**Table 1:** Powder filler materials usable in the PPTAW metal deposition of composite layers on selected structural materials.

Base Material	Powder Filler Material		Powder Grain (μm)	References
	Matrix	Reinforcement		
Structural steel	Ni–Cr–B–Si	69% WC/Co	53–106	[17]
Structural steel	Ni–base alloy	30% Cr_3_C_2_	75–185	[18]
Structural steel	Ni–base alloy	NbC	50–150	[19]
Structural steel	Fe–Cr–C–Ni	Chromium (II) carbide Cr_3_C_2_, Cr_7_C_3_, and Cr_23_C_6_	70–140	[20]
Structural steel	Fe–C–B–Mn–Si	20% B_4_C	50–150	[21]
Structural steel	Co–Cr–W–C	60% TiC+PD-W	60–250	[22]
Structural steel	Co–Cr–W–C	30% Cr_3_C_2_	60–145	[23]
Stainless steel	Co–Cr–W–C	50% Cr_3_C_2_, 20% WC, 50% TiC, 40% NbC	53–180	[24]
Stainless steel	Co–Mo–Cr–Si–Fe–Ni	35% WC 35% (WC−12% Cr)	53–180	[25]
Aluminium	Al–Ni	Al_2_O_3_, SiC, TiC	70	[26]
Titanium	Ti	50% NbC	80–120	[16]

**Table 2:** Chemical composition of AISI 4715 low-alloy structural steel according to manufacturer data (TimkenSteel Ltd., Canton, OH, USA) and chemical composition of Ni3+ WC-W_2_C-Co+PD-W powder.

**Chemical Composition of AISI 4715 Low-Alloy Structural Steel, wg. %**									
**C**	**Mn**	**S**	**P**	**Si**	**Cr**		**Mo**	**Ni**	**Fe**
0.12–0.18	0.65–0.95	≤0.015	≤0.015	0.15–0.35	0.40–0.70		0.45–0.60	0.65–1.00	Bal.
**Chemical composition of the Ni3 alloy, wg. %**							**Ceramic reinforcement of the matrix, wg. %**		
**C**	**Si**	**Mn**	**Cr**	**B**	**Fe**	**Ni**	**SFTC**	**FTC**	**PD-W**
≤0.05	2.4	0.5	2.0	≤1.4	≤0.5	Bal.	70	10	20

Carbide to matrix ratio, 60/35 (wg. %); bulk density of the powder determined by pycnometry, 11.64 g/cm^3^.

**Table 3:** Welding parameters of the plasma transfer arc welding (PTAW) metal deposition of the surface layer (Ni3+WC-W_2_C+PD-W composite powder) on steel AISI 4715.

Process Parameters	Value of Parameter
Current, I (A)	80
Voltage, U (V)	25
Travel speed, S (mm/s)	3
Powder feed rate, q (g/min)	18
Heat input, E_u_^(1)^ (J/mm)	400

Notes: Argon 5.0 (99.999%) acc. ISO 14175—I1: 2009 was used as plasma gas (flow rate = 1.6 L/min), Argon/Hydrogen 5% H2, Ar (welding mixture ISO 14175-R1-ArH-5) was used as shielding gas (flow rate = 12 L/min) and carrier gas (flow rate = 4 L/min), ^(1)^ calculated acc. to the formula: E_u_ = k∙(U × I)/S. The thermal efficiency coefficient for plasma transferred arc k = 0.6 was used.

**Table 4:** Results of the X-ray qualitative phase analysis of the surface layer (PPTAW metal deposition method, Ni3+WC-W_2_C+PD-W composite powder) deposited on structural low-alloy steel AISI 4715.

ICSD Card No	Phase Name	Chemical Formula	Crystalline Structure
98-007-7568	Tungsten carbide (2/1)	W_2_C	Hexagonal (P 63/m m c)
98-026-0166	Tungsten carbide (1/1)	WC	Hexagonal (P $\bar{6}$ m 2)
98-026-0172	Nickel	Ni	Regular (F m $\bar{3}$ m)
98-002-4306	Nickel boride (3/1)	Ni_3_B	Orthorhombic (P n m a)

**Table 5:** Density-related measurement results and calculations concerning the porosity of the surface layer (PPTAW metal deposition method, Ni3+WC-W_2_C+PD-W composite powder) deposited on structural low-alloy steel AISI 4715.

Physical Quantity	Average Value of the Measured Quantity
Density ρ (measured using the Archimedes method), g/cm^3^	9.3425
Absorbability A, %	1.3856
Open porosity P_o_, %	11.2421
Closed porosity P_c_, %	0.0082
Apparent density ρ_a_, g/cm^3^	8.9729
Total porosity P_c_, %	11.2503

**Table 6:** Hardness (HV10) measurement results concerning the external surface of the surface layer (PPTAW metal deposition method, Ni3+WC-W_2_C+PD-W composite powder) deposited on structural low-alloy steel AISI 4715 and the surface of the reference material (abrasion-resistant steel AR400).

Hardness (HV10)								
Specimen Designation	Specimen Number	Measurement Point Number					Average Hardness of the Tested Samples	Average Hardness of the Tested Materials
		1	2	3	4	5		
Ni3+WC-W_2_C+PD-W	N 01	634	746	883	545	733	708.2	702.5
	N 02	615	686	773	746	664	696.8	
AR400 Steel	S 01	430	421	420	429	424	424.8	424.1
	S 02	421	424	422	421	429	423.4	

**Table 7:** Results of the metal–mineral abrasive wear resistance tests concerning the surface layer (PPTAW metal deposition method, Ni3+WC-W_2_C+PD-W composite powder) deposited on structural low-alloy steel AISI 4715 in comparison with the abrasive wear resistance of abrasion-resistant steel AR400.

Specimen Designation	Spec. Number	Mass Before Test, g	Mass After Test, g	Mass Loss, g	Average Mass Loss, g	Clad Layer Density, g/cm^3^	Average Volume Loss, mm^3^	Relative ^(1)^ Abrasive Wear Resistance
Composite Coating								
Ni3+WC-W_2_C +PD-W	N 01	173.2112	173.0283	0.1829	0.1894	9.3425	20.2730	11.05
	N 02	162.8753	162.6794	0.1959				
Reference Material								
AR400 Steel	S 01	123.9290	122.2067	1.7223	1.7429	7.7836	223.9195	1
	S 02	121.7386	119.9752	1.7634				

Note: ^(1)^ relative abrasive wear resistance to Abrasion-Resistant Steel type 400.

## Data Availability

The data are not publicly available due to initiation of a patent procedure No. P435997.
